# Design and Experimental Study of a Current Transformer with a Stacked PCB Based on B-Dot

**DOI:** 10.3390/s17040820

**Published:** 2017-04-10

**Authors:** Jingang Wang, Diancheng Si, Tian Tian, Ran Ren

**Affiliations:** 1 State Key Laboratory of Power Transmission Equipment and System Security and New Technology, Chongqing University, Chongqing 400044, China; jingang@cqu.edu.cn; 2 Chongying Electric Power Design Institute, Chongying 401121, China; pperfecty@163.com (T.T.); ranranxinxing@126.com (R.R.)

**Keywords:** non-contact, magnetic field coupling, current transformer, current monitoring

## Abstract

An electronic current transformer with a B-dot sensor is proposed in this study. The B-dot sensor can realize the current measurement of the transmission line in a non-contact way in accordance with the principle of magnetic field coupling. The multiple electrodes series-opposing structure is applied together with differential input structures and active integrating circuits, which can allow the sensor to operate in differential mode. Maxwell software is adopted to model and simulate the sensor. Optimization of the sensor structural parameters is conducted through finite-element simulation. A test platform is built to conduct the steady-state characteristic, on-off operation, and linearity tests for the designed current transformer under the power-frequency current. As shown by the test results, in contrast with traditional electromagnetic CT, the designed current transformer can achieve high accuracy and good phase-frequency; its linearity is also very good at different distances from the wire. The proposed current transformer provides a new method for electricity larceny prevention and on-line monitoring of the power grid in an electric system, thereby satisfying the development demands of the smart power grid.

## 1. Introduction

In a power system, the accurate measurement of power-frequency current is an important approach in ensuring the reliable operation of a power distribution network. The accuracy, convenience, and speed of current transformers play a significant role in electric energy measurement, system monitoring and diagnosis, and power system fault analysis in smart power grids. Given that traditional electromagnetic current transformers (CTs) contain an iron core, their ferromagnetic effect tends to produce linear saturation and ferromagnetic resonance problems as well as insulation and volume and bandwidth response problems [1]. The method of current measurement by using Rogowski coil has been proposed [2,3], resulting in high measurement accuracy and good transient response. However, this kind of method based on the Rogowski coil targets high-frequency lightning current measurements, pulse current measurements, and partial discharge measurements on power cables, and the current loop to be measured must pass through the coil [4,5,6,7,8,9,10,11,12,13].

Current measurement methods based on the B-dot principle have been studied [14,15,16], resulting in high measurement accuracy and bandwidths reaching MHz. A B-dot coil is actually a special type of Rogowski coil that calculates the wire current via retroduction by sensing the change of magnetic field generated by the current-carrying wire. Compared with a normal Rogowski coil, a B-dot coil does not need to pass through the coil [17]. Most studies based on the B-dot principle focus on high-frequency current measurement (MHz, kA level) by a sensor under the self-integral state, such as in a lightening current and plasma discharge pulse current [18,19,20,21]. In comparison, only a few studies have examined the performance of B-dot sensors in measuring currents in differential mode.

In the paper, a B-dot sensor is proposed based on magnetic field coupling, and a non-contact electronic current transformer with stacked printed circuit board (PCB) is proposed. By analyzing the working principle and the working mode of the B-dot sensor and its influencing factors, a method for differential input and multiple electrode series-opposing is introduced, with the aim of designing a transformer that can be operated in differential mode for measuring the power frequency current of power system. The proposed transformer has the following advantages: good phase-frequency characteristic, good linearity, simple structure, and easy installation. It offers an alternative for electricity larceny prevention and on-line monitoring of a power grid in an electric system and meets the development requirements of smart power grids.

## 2. Principle of Current Measurement

### 2.1. Principal for B-Dot

**Figure 1:**
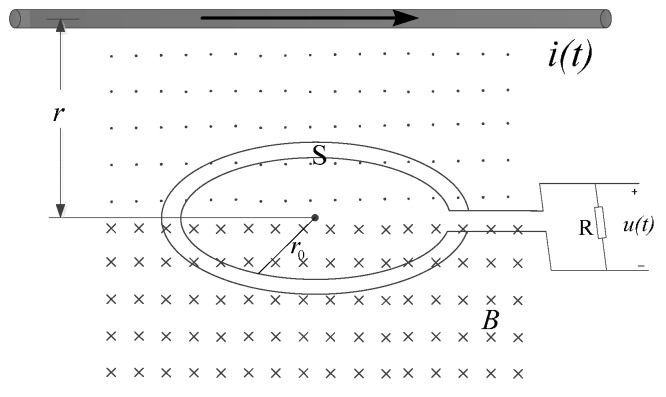
B-dot coil magnetic field induction.

The B-dot probe is a Rogowski coil with a special structure, and measures the current indirectly by measuring the changing magnetic field built by the changing current [17]. The principle for the B-dot current measurement is shown in Figure 1.

Here, *r* is the distance from the plane of the probe to the conductor, *r*_0_ is the radius of the probe coil, *S* is the area enclosed by the probe coil, *B* is the magnetic induction intensity generated by the current at a given moment, and *R* is a pure resistance.

According to the law of electromagnetic induction, when *r*_0_ *<< r*, the induced probe voltage in the magnetic field is given by

(1) $$e(t)=-\frac{d\psi (t)}{dt}=-\iint \frac{\mu_{0}}{2\pi r}dS\frac{di(t)}{dt}$$

To make $M=\iint \frac{\mu_{0}}{2\pi r}dS$, then

(2) $$e(t)=-M\frac{di(t)}{dt}$$

In Equation (1), coil magnetic flux is *ψ*, *S* is the active area of the probe coil, and *r* is the distance from probe center to the electrified wire. From Equation (2), the output voltage of the probe is proportional to the differential wire current. Thus, by calculating the integral of the induced electromotive force of the probe, the wire current can be calculated.

### 2.2. Sensor Design

**Figure 2:**
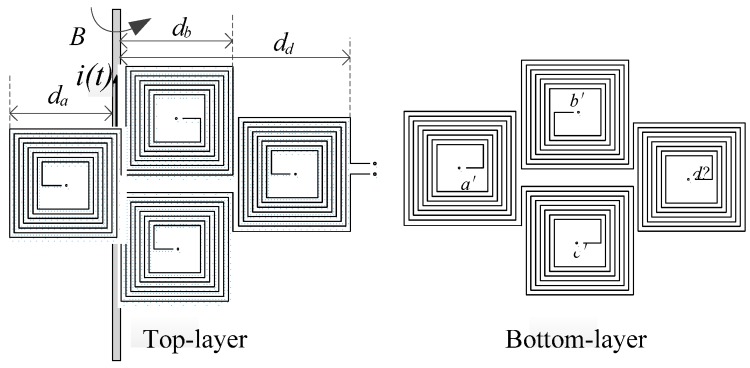
Series-opposing structure of the printed circuit board (PCB) air-core coil.

Based on the principle of B-dot coil current measurement, the PCB air-core coil sensor is designed. Its structure is shown in Figure 2. The top layer coil is reversely connected in series with the corresponding bottom coil through vias, and a total of eight air-core coils are placed successively in a series to form a loop. Obviously, the electromotive force of the coils is superimposed in sequence. By applying the Rogowski coil principle, the returning turn coils can eliminate the interference magnetic field from the external environment [22]. In the present paper, the idea of the returning turn design is adopted. Hence, the *d*, *d*’ coils are adopted as compensating coils. When the sensor is subject to external disturbance in the magnetic field, the *d* coil can counteract the interference flux in the *a*, *b*, *c* coils to a certain extent.

**Figure 3:**
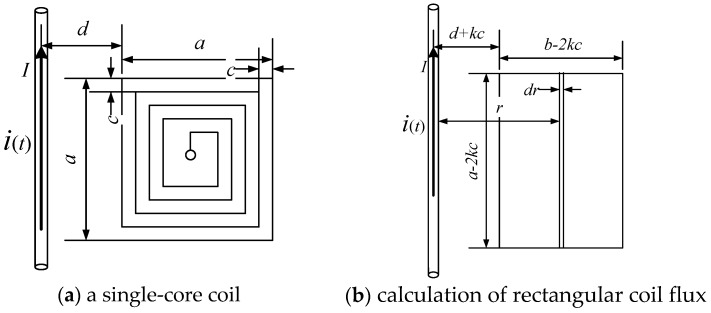
Principle of magnetic induction in a single-core coil.

As shown in Figure 3a, an air-core coil is equivalent to N square coils. Here, *r* is the distance from the point on the coil to the wire, *a* is the width of the outermost layer of the air-core coil, *c* is the distance between the turns of the coil, *d* is the distance from the outermost coil to the center of the wire, and *N* is the number of turns of a single air-core coil. In Figure 2, the distances of each air-core coil to the wire are given by *d_a_*, *d_b_*, *d_c_*, *d_d_* respectively, where *d_b_* is equal to *d_c_*.

According to the law of electromagnetic induction, the flux of a single turn coil in Figure 3b is given by

(3) $$\psi (t)=\int_{S}B(t)dS\;=\int_{d+kc}^{d+b-kc}\frac{\mu_{0}i(t)}{2\pi r}(a-2kc)dr$$

The total flux of a single air-core coil is expressed as

(4) $$\Phi (t)=\sum_{k=0}^{n-1}\psi =\sum_{k=0}^{n-1}\frac{\mu_{0}i(t)}{2\pi }(a-2kc)\ln(\frac{d+b-kc}{d+kc})\;e(t)=-\frac{d\Phi (t)}{dt}$$

From the mathematical relationship, we know that when *d* = 0, the flux Φ will reach the maximum value, so the induced voltage is the maximum value.

Induced electromotive force of PCB air-core coil is given by

(5) $$e(t)=e_{a}(t)+e_{b}(t)+e_{c}(t)-e_{d}(t)$$

(6) $$e(t)=-\sum_{k=0}^{n-1}\frac{\mu_{0}i(t)}{\pi }(a-2kc)\left\{\ln(\frac{d_{a}-kc}{d_{a}-a-kc})+2\ln(\frac{a+d_{b}-kc}{d_{b}+kc})-\ln(\frac{a+d_{d}-kc}{d_{d}+kc})\right\}$$

### 2.3. Sensor under Self-Integrating Mode

**Figure 4:**
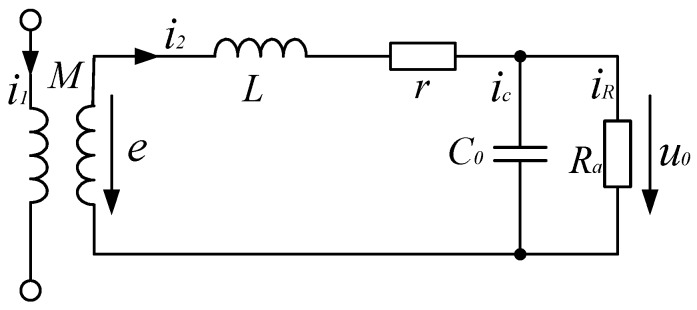
Equivalent circuit of the sensor under self-integrating mode.

As shown in Figure 4, when the value of sample resistance *R_a_* is small and 1/*ωC*_0_ >> *R_a_*, then *i_c_* ≈ 0, and *i_R_* ≈ *i*_2_, and when the current change rate is larger, namely, when *di*_2_/*dt* is quite large, we have

(7) $$M\frac{di_{1}}{dt}\approx L\frac{di_{2}}{dt}$$

Then the sample resistance voltage is expressed as

(8) $$u_{0}\approx i_{2}R_{a}=\frac{M}{L}i_{1}R_{a}$$

Now, the output voltage of the sample resistance is in direct proportion to the measured current; here, coil self-inductance L serves as an integral. Hence, the external integrating circuit is not required, because the circuit itself achieves integral function. Such a status is called self-integrating status [11,13,14]. Under this status, the sensor is suitable for the measurement of high-frequency current rather than a low-frequency current.

### 2.4. Sensor under Differential Mode

When *R_a_* is large (*R_a_* >> *r*) and the variation of *di*_2_/*dt* is small (relatively low-frequency), the PCB air-core coil equivalent circuit is similar to a pure resistance circuit. At this time, the sensor output voltage is given by

(9) $$u_{1}(t)\approx -M\frac{di_{1}(t)}{dt}$$

At this moment, an integrating circuit must be connected to the sensor so that the output voltage and measured current can have a direct ratio with each other. This kind of working condition is called differential status or external integral status.

**Figure 5:**
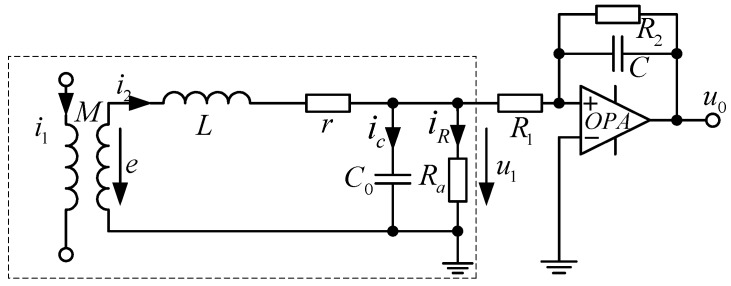
Equivalent circuit of the sensor with active integral.

In Figure 5, the transfer function in the measurement link is expressed as

(10) $$G_{1}\left(s\right)=\frac{U_{1}\left(s\right)}{I_{1}\left(s\right)}=\frac{Ms}{LC_{0}s^{2}+\left(\frac{L}{R_{a}}+rC_{0}\right)s+\left(\frac{r}{R_{a}}+1\right)}$$

The active integral link is given by

(11) $$G_{2}\left(s\right)=\frac{u_{0}\left(s\right)}{u_{1}\left(s\right)}=-\frac{R_{2}}{R_{1}}\frac{1}{R_{2}Cs+1}$$

The transfer function of the whole system is given by

(12) $$G\left(s\right)=G_{1}\left(s\right)\cdot G_{2}\left(s\right)=-\frac{\frac{MR_{2}}{R_{1}}s}{\left(R_{2}Cs+1\right)\left[LC_{0}s^{2}+\left(\frac{L}{R_{a}}+rC_{0}\right)s+\left(\frac{r}{R_{a}}+1\right)\right]}$$

**Figure 6:**
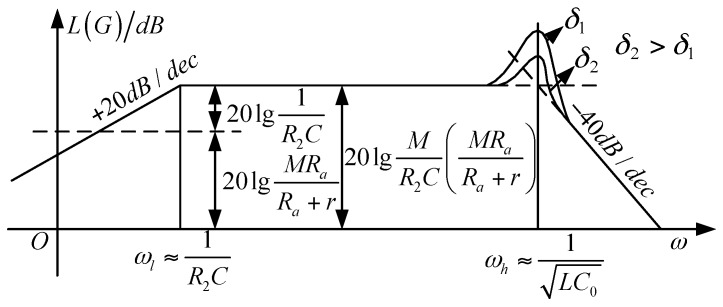
Amplitude-frequency characteristic of a sensor with an active integrator (lg is a base—10 logarithm).

The transfer function is required to draw a Bode figure, as shown in Figure 6.

Based on *R_a_* >> *r*, the system’s upper and lower cut-off frequency are simplified respectively as

(13) $$\{\begin{array}{c} f_{l}\approx \frac{1}{2\pi R_{2}C}\\ f_{h}\approx \frac{1}{2\pi \sqrt{LC_{0}}} \end{array}$$

The frequency band is given by

(14) $$\Delta f=f_{h}-f_{l}=\frac{1}{2\pi \sqrt{LC_{0}}}-\frac{1}{2\pi R_{2}C}$$

In Equation (13), the lower cut-off frequency of the system is determined by the feedback resistance *R*_2_ and the integral capacitance *C*, whereas the upper cut-off frequency is determined by the coil parameters.

The system sensitivity is given by

(15) $$S=\left|G\left(j\omega \right)\right|=\frac{MR_{2}}{R_{1}}\frac{1}{R_{2}C}=\frac{M}{R_{1}C}$$

In Equation (14), the sensitivity of the system can be improved by reducing *R*_1_, and the lower cut-off frequency can be reduced by increasing the *R*_2_. Therefore, the integral link can be utilized to properly adjust the cut-off frequency. Using the Bode function of MATLAB software to analyze the transfer function of the system, we find that the upper cut-off frequency of the sensor can reach MHz, and the lower cut-off frequency is close to 0 Hz. Therefore, when the sensor is operating in differential mode, it can meet the requirements of low frequency current measurement, which is suitable for measuring and monitoring power frequency current.

### 2.5. Anti-Interference Sensor Analysis

Interference magnetic field can be decomposed into two components: parallel to the PCB board and perpendicular to the PCB board.

#### 2.5.1. Magnetic Field Component Parallel to the PCB Board

**Figure 7:**
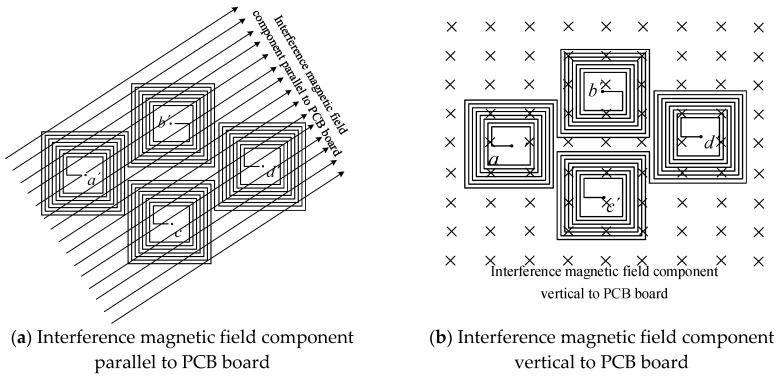
Influence of the external interference magnetic field.

When the magnetic field is parallel to the PCB board, as shown in Figure 7a, the direction of the magnetic field is parallel to the air-core coils, and the magnetic flux passing through these coils is zero; hence, no induced electromotive force is generated.

#### 2.5.2. Magnetic Field Component Vertical to the PCB Board

When the external magnetic field is a uniform magnetic field, the magnetic fluxes through the four air-core coils have the same size, and the induced electromotive forces are equal in size and opposite in direction. Due to the reverse series of the coils, the induced electromotive forces cancel each other out, as shown in Figure 7b. Therefore, the air-core coils can resist the interference of the uniform magnetic field.

**Figure 8:**
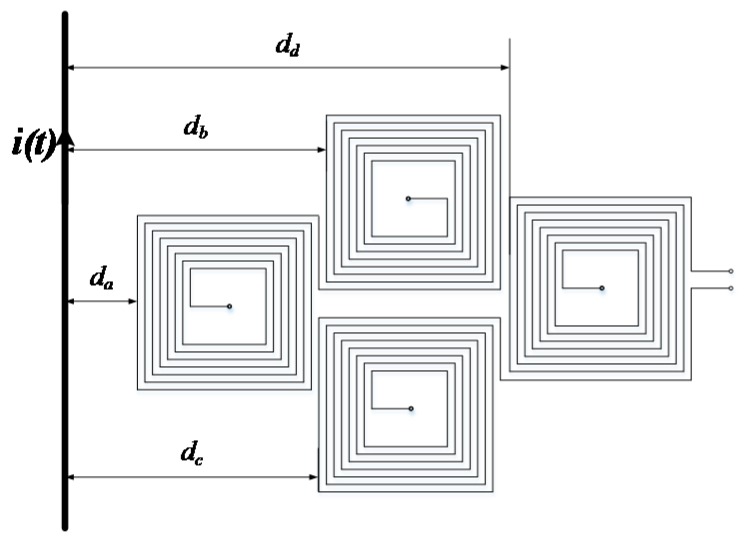
The calculations of interference magnetic.

The external interference current is taken as an example for this analysis. When the external magnetic field is not uniform, the current in the plane of the PCB board can produce a magnetic field perpendicular to the air-core coils. As shown in Figure 8, the interference current *i*(*t*) is located on the side of the PCB board. The distances of interference currents to each air-core coil are *d_a_*, *d_b_*, *d_c_* and *d_d_.* The length of the outer coil is *a*, the width is *b*, the turn pitch is *c*.

According to Equation (3),

(16) $$\Phi_{a}(t)=\sum_{k=0}^{n-1}\frac{\mu_{0}i(t)}{2\pi r}(a-2kc)\ln(\frac{d_{a}+a-kc}{d_{a}+kc})$$

(17) $$\Phi_{b}(t)=\sum_{k=0}^{n-1}\frac{\mu_{0}i(t)}{2\pi r}(a-2kc)\ln(\frac{d_{b}+a-kc}{d_{b}+kc})$$

(18) $$\Phi_{c}(t)=\sum_{k=0}^{n-1}\frac{\mu_{0}i(t)}{2\pi r}(a-2kc)\ln(\frac{d_{c}+a-kc}{d_{c}+kc})$$

(19) $$\Phi_{d}(t)=\sum_{k=0}^{n-1}\frac{\mu_{0}i(t)}{2\pi r}(a-2kc)\ln(\frac{d_{d}+a-kc}{d_{d}+kc})$$

The total flux generated by the interference current in the air-core coil is

(20) $$\Phi (t)=2[\Phi_{a}(t)-\Phi_{b}(t)-\Phi_{c}(t)+\Phi_{d}(t)]$$

**Figure 9:**
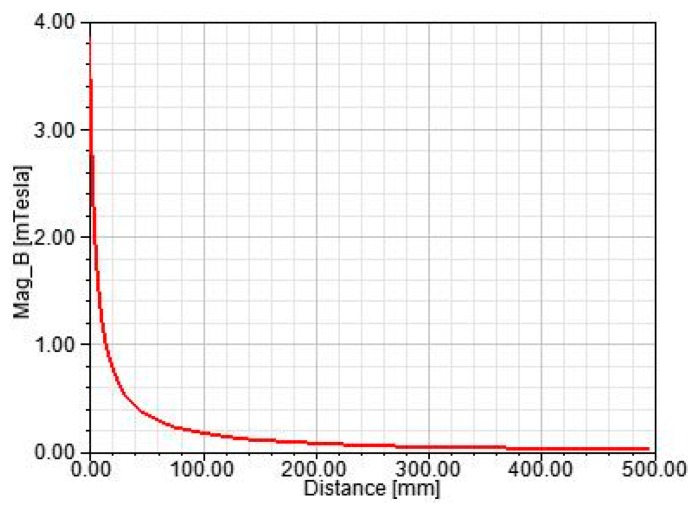
Magnetic field curve around the wire in 100 A.

In Figure 9, the magnetic field intensity decays rapidly along the radial direction of the wire, and at a distance of 0.5 m from the wire, it decays to about 3%. The influence of the magnetic field generated by other wires can be ignored when the other wires are away from the wire to be measured by more than 0.5 m. The transmission lines are usually overhead lines in the operation of the power system, and the wire spacing is much larger than 0.5 m. Therefore, the measurement accuracy can be achieved as long as the distance is maintained. Once the PCB air-core coil sensor is applied in more complex scenes, the adjacent phase interference must be considered.

## 3. Design of the Current Transformer

### 3.1. Sensor Optimization Based on Finite Element Simulation

The Ansoft Maxwell software is used to build the model of the conducting wire. The wire radius is set to 2.5 mm, and the current is set to 100 A. The variation curve of the magnetic field intensity around the wire is shown in Figure 9. As can be seen, the magnetic field intensity decays rapidly along the radial direction of the wire. At the distance of 0.5 m from the wire, it decays to about 3% of the maximum value. Therefore, when the current is measured by the sensor, the measuring points should be in the vicinity of the conductor region.

**Figure 10:**
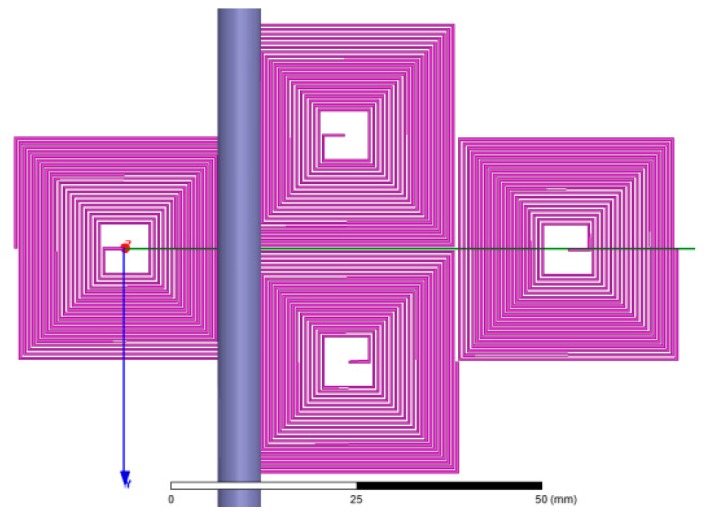
Finite element calculation model.

**Figure 11:**
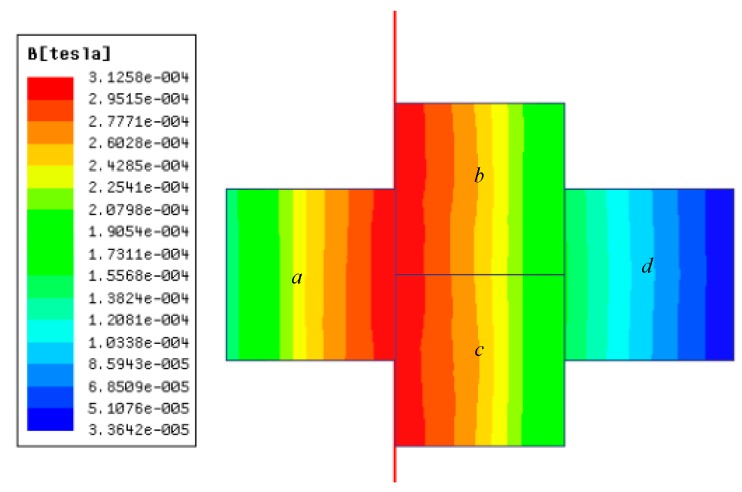
PCB air-core coil magnetic simulation calculation.

According to Equation (7), major factors influencing the sensor mutual induction coefficient are coil length, distance between turns, and the number of turns. Coil length and number of turns can influence the PCB size, whereas the distance between turns can affect the frequency response of the sensor. Finite element in Ansoft Maxwell software is applied to optimize the above parameters and guarantee proper mutual induction coefficient. The simulation settings are as follows: connecting transmission wire of 5 m with current of 50 Hz and 100 A, wire radius of 2.5 mm, region size that is 300% of the calculation model, and border region calculation of 0 to simulate the situation wherein the magnetic field at the infinite point is 0, as shown in Figure 10. Based on the simulation, the model is further optimized and improved to obtain higher voltage output. The distribution of the magnetic field is shown in Figure 11.

The calculation of the magnetic flux density of the sensor coil is performed by using the Maxwell software. Then, the output voltage of the sensor is calculated based on the flux data obtained. After considering all the parameters and the output voltage, a set of suitable parameters is selected to determine the structure of PCB air-core coil. The coil parameters are shown in Table 1.

Based on the parameters, the total magnetic flux of the coil in *a*, *b*, *c*, *d*, which are obtained from the simulation are −2.28 × 10^−6^, −2.32 × 10^−6^, −2.31 × 10^−6^ and −5.04 × 10^−7^ Wb, respectively. Thus, the total magnetic influx of the CT based on PCB planar-type air-core coil is given by

$$\Phi =2\times \left(\psi_{a}+\psi_{b}+\psi_{c}-\psi_{d}\right)=2\times 6.41\times 10^{-6}=12.82\times 10^{-6}Wb$$

The mutual induction efficient is expressed as

(21) $$M=-\frac{\Phi }{I}.$$

The output voltage of the whole transformer is given by

$$e(t)=-M\cdot \frac{di(t)}{dt}=-M\cdot \frac{d100\sin(100\pi t)}{dt}\;\;\;\mathrm{Amplitude}:E=4.03\;\mathrm{mV}$$

**Figure 12:**
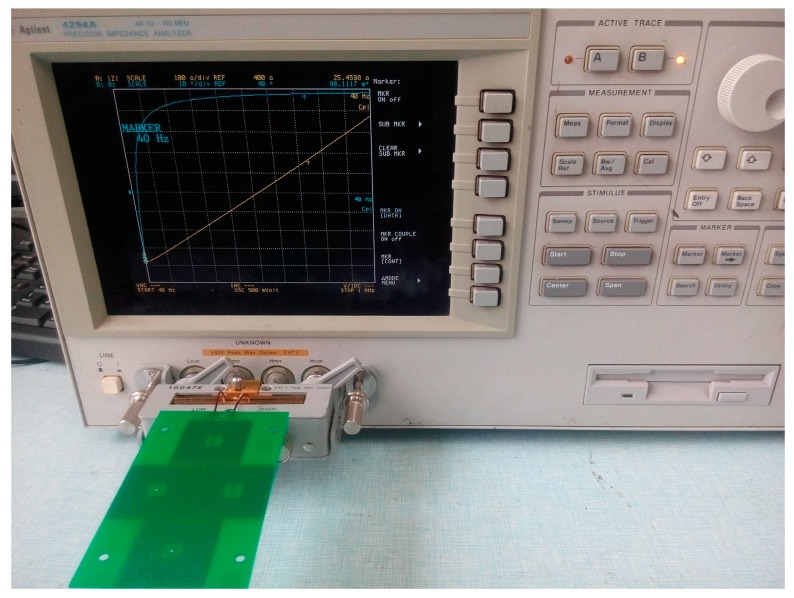
Parameter measurement of the PCB air-core coil sensor.

Finally, the PCB air-core coil sensor is fabricated by calculating the coil parameters [23]. The impedance of the sensor coil itself is measured by using Agilent 4294A (Agilent Technologies Inc., Santa Clara, CA, USA), as shown in Figure 12. Measurement is done within the scope of 40 Hz to 4 MHz. The induction and capacitance parameters have certain fluctuations. The result is shown in Table 2.

### 3.2. Device Design for the Electronic Transformer

**Figure 13:**
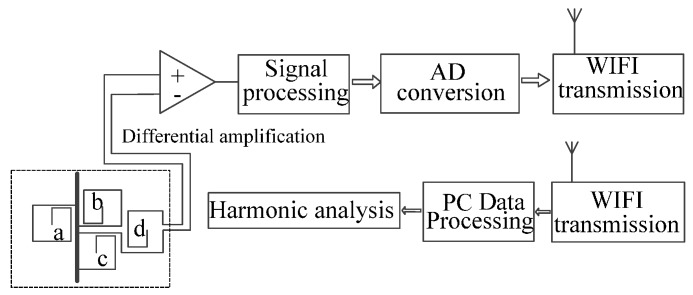
A model of the current transformer structure.

In consideration of the low output voltage of the single-block PCB sensor, multi-block PCBs are connected in a series to improve output voltage [24]. The transformer structure is designed as shown in Figure 13, and consists of the sensor, signal acquisition unit, and signal processing unit.

The output signal of the sensor is collected by a circuit with a differential amplifying structure [25,26]. The differential amplifier circuit is mainly composed of a differential amplifier AD620 (Analog Devices Inc., Norwood, MA, USA) and gain resistor. The AD620 performs a differential operation on the output signal to remove the common-mode signal with the same polarity in the interfering signal. Simultaneously, the differential mode signal with opposite polarity is amplified, and this differential mode signal is a useful signal we want to obtain. This not only increases the output of the transformer, but also restrains the common mode interference, such as high-frequency noise signal. 

For the integral link, an active integrator with an inertial element acts as a circuit for restoring the original signal. When measuring the current of the high-voltage transmission line, as an electronic transformer, introducing the AD module and WIFI technology is convenient, after which the data is sent to the remote terminal using WIFI technology.

## 4. Test Result Analysis

### 4.1. Test Platform

**Figure 14:**
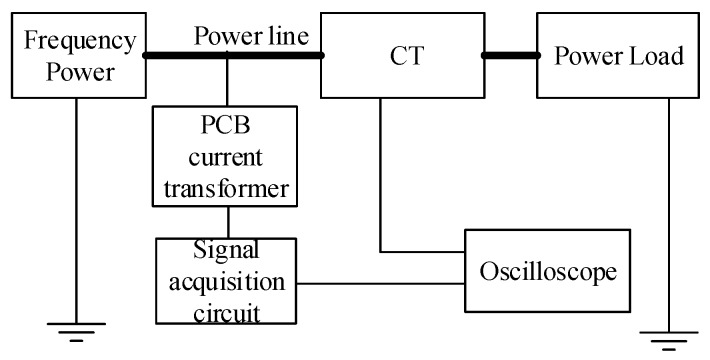
Test platform structure.

A current test platform is built to simulate the current measurement environment of the power distribution network. The structure of the platform is shown in Figure 14. Both the traditional electromagnetic current transformer (CT, HL-3, 0.2 level) [27], and the designed transformer are used at the same time to conduct current measurement for the power wire in the platform. Here, the WIFI technology is no longer needed because of the low power output.

**Figure 15:**
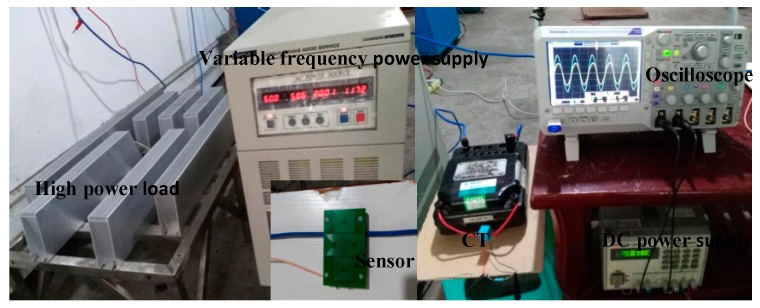
Test platform.

**Figure 16:**
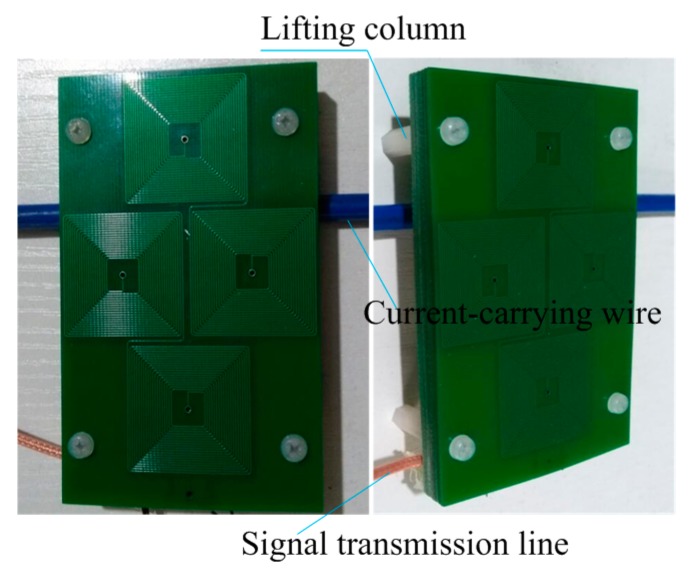
Sensor test physical picture.

The test platform is shown as Figure 15 and Figure 16; it uses the program-controlled convertor (type: JJ98DD53D, Shandong Jingjiu Science and Technology Co., Ltd., Jinan, China) as the power supply and the high-power resistance is considered the load. The oscilloscope (Tektronix, DPO2014B, Beaverton, OR, USA) is applied as a waveform display and measuring equipment. The CT is used as the measurement standard to realize the calibration of the designed transformer. The amplitude, phase position, and waveform are compared to verify the performance of the designed transformer.

### 4.2. Steady-State Performance Test

**Figure 17:**
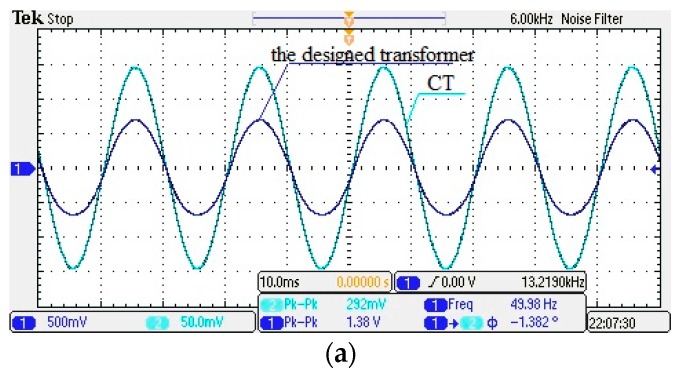

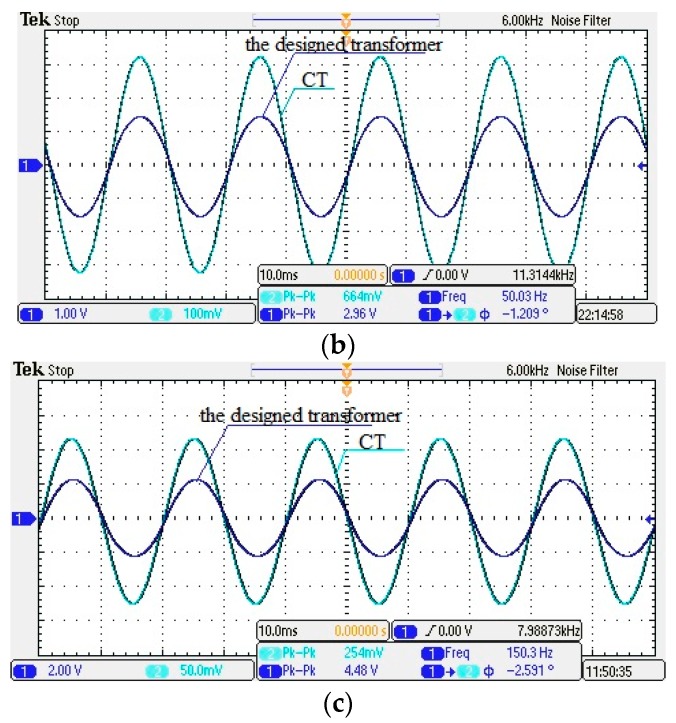
The steady state waveform of the designed transformer and the current transformer (CT). (**a**) *f* = 50 Hz, *I* = 10 A, *D* = 5 mm; (**b**) *f* = 50 Hz, *I* = 23 A, *D* = 5 mm; (**c**) *f* = *1*50 Hz, *I* = 9 A, *D* = 5 mm.

On the above test platform, the sensor is fixed above the wire, and the vertical relative distance D is kept invariant, as shown in Figure 14. The current output is adjusted gradually. In addition, the secondary CT is connected to a resistive load for I-V conversion. The output waveform of the designed transformer and the CT is displayed via oscilloscope, as shown in Figure 17. The oscilloscope channel 1 shows the output waveform of the designed transformer, whereas channel 2 shows the CT output waveform.

In Figure 17, the output waveform of the transformer is basically the same as that of the CT, which indicates that the transformer has good steady-state response characteristics. If *φ* < 0, the phase of the designed transformer’s output voltage is delayed.

As stipulated in IEC60044-8-2002 Standard [28], ratio error *ε*% and phase position difference *φ* are respectively defined as

(22) $$\varepsilon \%=\frac{K_{n}U_{s}-I_{p}}{I_{p}}\times 100\%,$$

(23) $$\phi =\phi_{s}-\phi_{p}.$$

Here, *U_s_* is the output voltage of the designed transformer, *K_n_* is the rated transformation ratio, *I_p_* is the current in the wire, *φ_p_* is the phase position of the designed transformer, and *φ_s_* is the phase position of the standard CT.

First, we adjust the power to current changes between 0 A and 40 A; then, when the output current changes, the amplitude and frequency of both the CT and the transformer output voltage are recorded, and the waveform is saved. Finally, the experimental data are collated, and the ratio difference and phase difference of the transformer are calculated. The results are shown in Table 3. In this Table, *I_n_* is the rated current, *I_m_* is the wire current, and *U_p_* is the output voltage of the transformer.

The results above show the following:

(1) In Table 3, within the rated current range from 2% to 120%, the transformer ratio error *ε*% < 0.9%, and the phase difference *φ* < 3°. Thus, the designed transformer has high measurement accuracy.
(2) In Figure 17 and Table 3, a small error can be found in the phase position, which may be due to the parasitic capacitance between the coils and the resulting coil inductance.

### 4.3. Linearity Test

On the test platform, the linearity performance is tested. The test is done via three steps: (1) adjust the distance from the sensor (PCB board) to the wire, taking 5 mm as a unit (change the height of lifting column in Figure 16); (2) gradually adjust the output current from 0 A to 40 A; and (3) record the data. The experimental data when the distance D is 5 mm are shown in Table 4.

**Figure 18:**
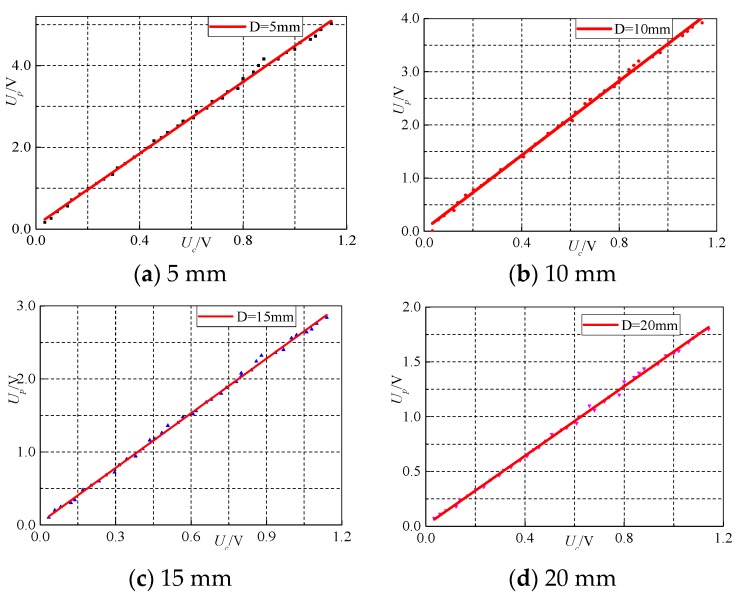

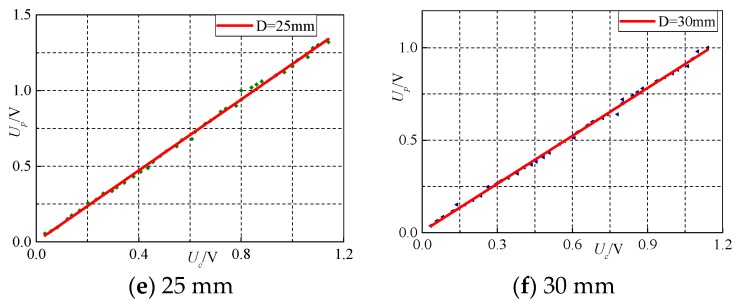
Curve fitting of current at different distances

In fitting the experimental data of transformer and CT, the horizontal axis represents the output voltage of the transformer, whereas the vertical axis represents the CT. The fitting curve is shown in Figure 18. The ratio of the linear fitting curve is the correction factor of the transformer error.

Moreover, linearity is tested when *D* has the values of 10, 15, 20, 25, and 30 mm. The data are shown in Table 5.

The test results show the following:

(1) As shown in Figure 18, each point is extremely close to the fitting line, which indicates good linearity of the designed transformer.
(2) In Table 5, the designed transformer maintains a small phase error at different distances. However, as can be seen in Table 5, the first-order fitting correction factor with the CT is gradually reduced. This finding means that when the distance from the designed transformer to the wire becomes closer, the transformer linearity also improves. However, as the distance increases, the phase error tends to enlarge. Chances are that when the distance is closer, the magnetic induction line through the sensor is denser, and then the impact of interference on the environment surrounding the sensor becomes relatively weak. When the distance is far, the sensor becomes more susceptible.

### 4.4. On-Off Operation Test

On the test platform, the transient performance of the designed transformer is tested under the on-off state. The sensor is fixed above the wire, and the relative position remains constant. Under different current levels, the power is switched off suddenly so as to cut off the current, then the oscilloscope is used to record the waveform response of voltage attenuation for both the designed transformer and the CT.

**Figure 19:**
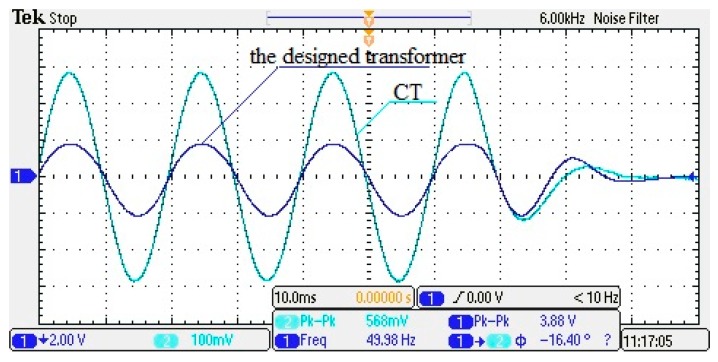
Switching transient waveform (*I* = 30 A, *D* = 5 mm, *f* = 50 Hz).

In Figure 19, the results show that the output waveform of the transformer closely follows the change of current, and its output voltage is reduced within a certain period to 10% of the peak before the fault in a very short period of time. Furthermore, the waveform no longer fluctuates. Therefore, the designed transformer can effectively reflect and track the changes in the current in the wire.

### 4.5. Frequency Test

**Figure 20:**
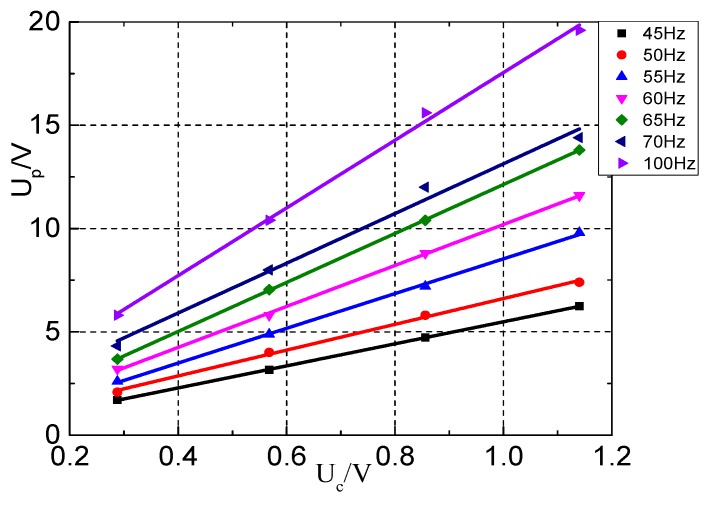
Voltage fitted curve under different frequencies

This experiment is done to verify the performance of the transformer under different frequencies. According to the analysis of the test data of the linearity of the transformer, the smaller distance *D* has a smaller phase error, so the selected distance for testing is set to is 5 mm. The corresponding data linear fitting is shown in Figure 20.

In Figure 20, the output voltage of the designed transformer has good linearity under different frequencies. This finding means that the transformer has good frequency response under low frequency. Limited by the output power and frequency of the power supply, there is no longer a need to conduct more performance tests of the designed transformer at higher current and higher frequency.

## 5. Conclusions and Future Prospects

In this paper, by studying the relationship between the current in the wire and its surrounding magnetic field, a non-contact electronic current transformer based on the magnetic field coupling principle is proposed. According to the experimental results, compared with the traditional CT, when the designed transformer is used to measure the power frequency current, the results demonstrate that it has very good steady-state and transient characteristics as well as high accuracy. At the same time, the results show that it has excellent linearity when the measuring distance is changing. In addition, the current transformer has good accuracy and small phase error.

In further studies, more performance tests of the designed transformer shall be carried out at higher currents and higher frequencies. To improve the anti-interference capacity of the sensor, research on phase compensation to the sensor will also be done, along with an investigation into the structural optimization of the sensor.

## Figures and Tables

**Table 1:** Design parameters for single-layer PCB air-core coil structure.

**Coil**	**Number of Turns (n)**	**Length (cm)**	**Electrode Width (mil)**	**Distance Between Electrodes (mil)**
	4 × 25	38	8	8

**Table 2:** Inner impedance parameter of PCB air-core coil.

**PCB Air-core coil**	**Resistance**	**Induction**	**Fluctuating Deviation**	**Capacitance**	**Fluctuating Deviation**
	25.75 Ω	149.36 μH	5 μH	37.2 pF	8 pF

**Table 3:** Accuracy measurement for the current transformer (*D* = 1 mm).

Measuring Point	*I_m_*/A	*U_p_*/mv	*ε*%(±)	*φ*/(°)
2% *I*_n_	0.602	60.62	0.70	2.0
5% *I*_n_	1.511	151.87	0.51	1.9
10% *I*_n_	3.024	303.62	0.40	1.6
20% *I*_n_	6.013	599.42	0.31	1.5
40% *I*_n_	12.161	1208.29	0.64	1.5
60% *I*_n_	18.06	1818.36	0.68	1.4
80% *I*_n_	24.120	2419.32	0.30	1.3
100% *I*_n_	30.060	3016.12	0.34	1.3
120% *I*_n_	36.042	3625.12	0.58	1.2

**Table 4:** Output voltage of the transformer under different currents (*D* = 5 mm, *f* = 50 Hz).

*U_c_*/mV	*U_p_*/mV	*I*/A	*U_c_*/mV	*U_p_*/mV	*I*/A
60	264	1.99	632	2880	22.01
121	568	4.01	680	3120	23.97
180	860	6.02	744	3360	26.01
226	1120	7.98	808	3600	27.99
292	1380	10.03	848	3840	30.02
352	1600	12.01	920	4160	32.02
396	1880	13.99	968	4320	34.02
464	2160	16.03	1020	4560	36.01
516	2360	17.97	1080	4720	37.99
580	2640	20.01	1140	5040	40.02

**Table 5:** Transformer fitting data under various distances.

Dstance/*D*	Fitting Correction Coefficient	Congruence Mean Error	Maximum Phase Difference φ (°)
5 mm	4.39	0.0041	1.5
10 mm	3.49	0.0027	2.0
15 mm	2.29	0.0017	2.1
20 mm	1.59	0.0026	2.5
25 mm	1.17	0.0032	2.8
30 mm	0.86	0.0018	3.2

## References

[1] Wang Z., Zhang X., Wang F., Lan X., Zhou Y. (2016). Effects of aging on the structural, mechanical, and thermal properties of the silicone rubber current transformer insulation bushing for a 500 kV substation. SpringerPlus.

[2] Yamamoto K., Ueda N., Ametani A., Natsuno D. (2012). A Study of Lightning Current Distribution at a Wind Turbine Foot: Influence on Current Measurements Using a Rogowski Coil. Electr. Eng. Jpn..

[3] Metwally I.A. (2016). Tape-wound Rogowski coil for measuring large-magnitude pulsed currents. Instrum. Exp. Tech..

[4] Ardila-Rey J.A., Albarracín R., Álvarez F., Barrueto A. (2015). A validation of the spectral power clustering technique (SPCT) by using a Rogowski Coil in partial discharge measurements. Sensors.

[5] Metwally I.A. (2010). Self-Integrating Rogowski Coil for High-Impulse Current Measurement. IEEE Trans. Instrum. Meas..

[6] Yutthagowith P., Pattanadech N., Kunakorn A., Leelajindakrairerk M. Design and construction of a Rogowski’s coil with compensated RC integrators for measuring impulse current. Proceedings of the International Power Engineering Conference (IPEC 2007).

[7] Liu Y., Lin F., Zhang Q., Zhong H. (2011). Design and construction of a Rogowski coil for measuring wide pulse current. IEEE Sens. J..

[8] Moonmirat P., Kunakorn A., Yutthagowith P. A wide bandwidth Rogowski coil with an active integrator for measurement of impulse currents. Proceedings of the Asia-Pacific International Conference on Lightning (APL 2013).

[9] Liu Y., Xie X., Hu Y., Qian Y., Sheng G., Jiang X. (2016). A novel transient fault current sensor based on the PCB Rogowski Coil for overhead transmission lines. Sensors.

[10] Shafiq M., Kutt L., Lehtonen M., Nieminen T., Hashmi M. (2013). Parameters Identification and Modeling of High-Frequency Current Transducer for Partial Discharge Measurements. IEEE Sens. J..

[11] Moreno M.V.R., Robles G., Albarracín R., Rey J.A., Tarifa J.M.M. (2016). Study on the self-integration of a Rogowski coil used in the measurement of partial discharges pulses. Electr. Eng..

[12] Marracci M., Tellini B. Analysis of precision Rogowski coil via analytical method and effective cross section parameter. Proceedings of the 2016 IEEE International Instrumentation and Measurement Technology Conference (I2MTC).

[13] Metwally I.A. (2015). Multi-layer self-integrating Rogowski coils for high pulsed current measurement. Instrum. Exp. Tech..

[14] Yao C., Xiao Q., Mi Y., Yuan T., Li C., Sima W. (2011). Contactless Measurement of Lightning Current Using Self-integrating B-dot Probe. IEEE Trans. Dielectr. Electr. Insul..

[15] Zhang H., Xia L., Shen Y., Li Q., Wang Y., Zhang L., Liu K. (2016). Analysis and Process of B-Dot Waveformsin a High-Current Injector. IEEE Trans. Plasma Sci..

[16] Huiskamp T., Beckers F.J.C.M., van Heesch E.J.M., Pemen A.J.M. (2016). B-Dot and D-Dot Sensors for (Sub)Nanosecond High-Voltage and High-Current Pulse Measurements. IEEE Sens. J..

[17] Ahmad A.A., Robert A.S. (2014). Calibration of Electromagnetic Dot Sensor—Part 2: B-dot Mode. IEEE Sens. J..

[18] Hardin R. (2012). Magnetic Field Generation and B-dot Sensor Characterization in the High Frequency Band. Master’s Thesis.

[19] Wagoner T.C., Stygar W.A., Ives H.C., Gilliland T.L., Spielman R.B., Johnson M.F., Reynolds P.G., Moore J.K., Mourning R.L., Fehl D.L. (2008). Differential-output B-dot and D-dot monitors for current and voltage measurements on a 20-MA, 3-MV pulsed-power accelerator. Phys. Rev. Spec. Top. Accel. Beams.

[20] Lane B., Campbell C., Sawada I., Ventzek P.L.G. (2016). Measurement of spatial and temporal evolution of electromagnetic fields in a 100 MHz plasma source using B-dot and double dipole probes. J. Vac. Sci. Technol. A.

[21] He J., Guo R. (2010). Measurement of arc velocity in a rotating-arc pulsed-power switch based on B-dot probes. IEEE Trans. Plasma Sci..

[22] Tao T., Zhao Z., Pan Q., Tang J., Zhang Y. (2011). Design of PCB Rogowski coil and analysis of anti-interference. Trans. Chin. Electrotech. Soc..

[23] Wang R., Prabhakaran S., Burdick W., Raymond N. Rogowski current sensor design and analysis based on printed circuit boards (PCB). Proceedings of the 2014 IEEE Energy Conversion Congress and Exposition (ECCE).

[24] Baschirotto A., Dallago E., Malcovati P., Marchesi M., Venchi G., Rossini A. Multilayer PCB Planar Fluxgate Magnetic Sensor. Proceedings of the 2006 Ph.D. Research in Microelectronics and Electronics (PRIME).

[25] Kubo T., Furukawa T., Itoh H., Hisao W., Wakuya H. Numerical electric field analysis of power status sensor observing power distribution system taking into account measurement circuit and apparatus. Proceedings of the SICE Annual Conference.

[26] Koziy K., Bei G., Aslakson J. (2013). A low-cost power quality meter with series arc-fault detection capability for smart grid. IEEE Trans. Power Deliv..

[27] (2006). GB 1208-2006, Current Transformers.

[28] (2002). IEC 60044-8-2002, Part 8: Electronic Current Transformers.

